# Tissue-resident immune cells in cervical cancer: emerging roles and therapeutic implications

**DOI:** 10.3389/fimmu.2025.1541950

**Published:** 2025-04-22

**Authors:** Xidie Li, Juan Deng, Xiaoping Liu, Yan Zhou, Tingting Bi, Jingjing Chen, Jinjin Wang

**Affiliations:** ^1^ Department of Obstetrics and Gynecology, Zhuzhou Central Hospital, Zhuzhou, Hunan, China; ^2^ Department of Breast Surgery, Zhuzhou Central Hospital, Zhuzhou, Hunan, China

**Keywords:** cervical cancer, tissue-resident immune cells, local tumor microenvironment, Trm cell differentiation, tissue-resident memory T cells

## Abstract

The favorable prognosis of “hot” tumors is widely acknowledged in oncology. Recently, the concept of tertiary lymphoid structures (TLS) has renewed appreciation for local immune cells within tumor tissues. Tissue-resident immune cells, a newly identified subset of tumor-infiltrating lymphocytes, are emerging as potential key players in tumor infiltration and TLS formation, due to their ability to reside indefinitely within tissues and mount effective responses to local antigens. Cervical cancer (CC), the fourth most common cause of cancer-related mortality among women globally, has experienced comparatively limited progress in delineating its tumor immune microenvironment compared to other malignancies. Notably, the role of tissue-resident immune cells within the CC milieu remains inadequately characterized. This comprehensive review aims to synthesize current knowledge and critically evaluate the putative roles of these cells in CC pathogenesis, providing new insights on the intricate dynamics of the local tumor microenvironment.

## 1 Introduction

Cervical cancer (CC) ranks as the fourth most common gynecological malignancy affecting women globally, representing a significant threat to women’s health (1). According to the GLOBOCAN 2020 report, there were an estimated 604,000 new CC cases worldwide, resulting in approximately 342,000 deaths (2). Despite advancements in multimodal treatment approaches, including surgery, radiotherapy, and chemotherapy, a considerable proportion of patients with advanced-stage CC experience tumor recurrence and poor clinical outcomes. The challenge of effectively reducing disease recurrence and improving long-term survival rates in CC patients remains a critical issue in gynecologic oncology.

The tumor microenvironment (TME) has emerged as a focal point for cancer therapy research, offering potentially safer and more efficacious therapeutic targets (3). Over the past decades, our conceptualization of tumors has evolved from viewing them as simple aggregates of abnormally proliferating cells to recognizing them as complex, highly organized “pseudo-organs”. The TME comprises a heterogeneous population of cells, including immune cells (such as CD8^+^ and CD4^+^T lymphocytes, B cells, and natural killer cells), stromal components (including cancer-associated fibroblasts, adipocytes, and extracellular matrix), and vascular elements (both blood and lymphatic endothelial cells). Among these constituents, immune cells have garnered particular attention due to their capacity to recognize and potentially suppress malignant cells, thus emerging as promising targets for immunotherapeutic interventions.

In recent years, the significance of regional immune responses—localized within specific tissues or organs and functioning independently of systemic circulation—has been increasingly recognized in various diseases. It has been observed that immune cells adapt to the microenvironment of tumor tissues, acquiring unique characteristics and functional specializations that may play critical roles in antitumor immunity. This systematic review examines the potential roles of resident immune cells in CC and offers perspectives on their targeting for tumor immunotherapy.

## 2 Tissue-resident immune cells: definition and subsets

TRICs are a specialized group of immune cells that reside long-term in specific tissues and perform localized immune functions. Unlike circulating immune cells, TRICs remain in tissues under steady-state or non-pathological conditions, establishing local immune memory and rapid response capabilities (4, 5). TRICs primarily include the following subsets:

### 2.1 Tissue-resident memory T cells

First identified in skin and intestinal tissues, Trm cells were definitively characterized in 2009 through murine models. Trm cells comprise two primary subsets: CD8^+^ and CD4^+^Trm. CD8^+^Trm cells predominantly mediate immune responses in infection and tumor immunity, enabling rapid pathogen and tumor cell elimination (6). CD4^+^Trm cells have recently gained attention in autoimmune disease research, where they modulate immune responses through cytokine secretion and interactions with other immune cells (7, 8). While CD8^+^Trm cells focus on direct pathogen and tumor cell clearance, CD4^+^Trm cells play a regulatory role, supporting local immune responses and tissue repair processes.

### 2.2 Tissue-resident B cells

Tissue-resident B Cells (also known as resident memory B cells, Brm) represent a recently characterized subset of B lymphocytes, first comprehensively described in 2019 (9). Initially identified in barrier tissues such as the lungs and intestines, these cells have since been discovered in diverse anatomical locations, including the skin, liver, and tumor microenvironments (10). Brm cells exhibit distinct transcriptional and phenotypic profiles that enable long-term persistence within specific tissue compartments and rapid responses to pathogen re-encounter. Unlike circulating memory B cells, they possess unique capabilities for immediate local immune responses, rapidly differentiating into antibody-secreting cells and directly engaging antigens within the tissue microenvironment. Their primary functions encompass local antibody production, immune regulation, and critical contributions to host defense against various pathological conditions, including viral infections, bacterial challenges, autoimmune diseases, and tumor immunity, thereby providing a more targeted and expeditious immune defense.

### 2.3 Tissue-resident NK cells

Although the discovery of NK cells dates back to the 1970s, it is only in the past two decades that researchers have begun to focus on the tissue-resident characteristics of these cells (11). The tissue-resident NK cells were first identified in the liver of mice and have since been found in various human tissues, including the skin, salivary glands, uterus, adipose tissue, and kidneys (12, 13). These cells possess unique phenotypic and functional properties that enable them to rapidly respond to local infections, tissue damage, or tumors. Their localized presence allows tissue-resident NK cells to exert immediate immune responses through cytokine secretion and direct killing of infected or malignant cells, while also modulating local immune environments and maintaining tissue homeostasis.

## 3 Potential characteristic molecular markers of TRICs

Molecular markers serve as critical tools for defining and investigating tissue-resident immune cells. These markers not only facilitate the identification of distinct cellular subsets but also provide insights into their functional properties and tissue-specific adaptations. While the current understanding of molecular markers is primarily derived from studies on Trm cells, the functional implications of these markers may extend to other tissue-resident immune cell populations. Based on their established roles and potential functions, these molecular markers can be categorized into several groups (**Table 1**):

**Table 1:** Potential characteristic molecular markers associated with tissue-resident immune cells.

Category	Molecular Marker	Function	Role in Tissue-Resident Immune Cells
Tissue residency	CD103	Forms integrin αEβ7; binds E-cadherin on epithelial cells	Mediates retention of immune cells within epithelial tissues
	CD69	Early activation marker; downregulates S1PR1	Promotes tissue retention by attenuating the response to S1P gradients; deficiency leads to increased egress of immune cells into circulation
	CD49a	Forms VLA-1 with integrin β1; binds extracellular matrix proteins	Facilitates adhesion and residence within tissues by binding to collagen
	S1PR1/KLF2	S1PR1 mediates lymphocyte egress; KLF2 regulates S1PR1 expression	Reduces responsiveness to egress signals, promoting retention in peripheral tissues
Migration Adhesion	CD11a	Adhesion molecule; binds ICAM-1	Enhances localization and retention
	CD44	Adhesion molecule; binds hyaluronic acid	High expression enhances immune cell localization and adhesion within peripheral tissues
	CD62L	Mediates lymphocyte homing to lymphoid tissues	Expression levels influence tissue localization capabilities
	CXCR6/CCR7	CXCR6 binds CXCL16; CCR7 binds CCL19 and CCL21	High CXCR6 expression and low CCR7 expression promote immune cell migration to and retention within peripheral tissues
	CCR5/CCR6/CCR9/CCR10	Chemokine receptors guiding tissue-specific migration	Direct immune cells to specific tissues in response to chemokine gradients; contribute to localized immune responses
Transcription Factors	Hobit/Blimp-1	Regulate expression of tissue-residency genes	Facilitate differentiation and maintenance of Trm cells; suppress genes related to circulation
	Runx3	Enhances tissue-resident phenotype	Upregulates CD103 and promotes retention of immune cells
Inhibitory Receptors	PD-1/TIM-3/LAG-3/TIGIT	Inhibitory receptors modulating effector functions	Moderate expression prevents hyperactivation; impacts formation and persistence of memory cells
	KLRG1	Inhibitory receptor suppressing immune cell activity	Transition from KLRG1^+^cells to a tissue-resident phenotype indicates improved local immune response
Other Molecules	IL-15/IL-15Rα TGF-β/TGF-βR	IL-15 promotes proliferation and survival; TGF-β promotes differentiation	IL-15 essential for Trm formation; TGF-β signaling crucial for CD69 and CD103 upregulation and Trm retention
	RGS1	Negative regulator of chemokine receptor signaling	Limits responsiveness to chemokine gradients, maintaining cell tissue residency by reducing emigration

### 3.1 Molecules associated with tissue residency

The establishment and maintenance of tissue residency rely on several key molecular mechanisms. CD103 (Integrin αE), forming the heterodimeric integrin αEβ7, mediates epithelial tissue retention through E-cadherin binding (14). Its expression is indispensable for strategic positioning and long-term maintenance of TRICs within tissues. Similarly, CD49a (integrin α1) combines with integrin β1 to form VLA-1, facilitating adhesion to extracellular matrix components, particularly in organs such as the liver, skin, and female reproductive tract (15).

Tissue retention mechanisms critically involve the CD69-S1PR1 regulatory axis. CD69 promotes tissue residency by downregulating S1P receptor 1 (S1PR1), thereby attenuating cellular response to sphingosine-1-phosphate (S1P) gradients that typically guide lymphocyte egress (16–18). This process is regulated by Krüppel-like factor 2 (KLF2), a transcription factor that modulates S1PR1 expression. Studies have demonstrated that CD69 deficiency or elevated S1PR1/KLF2 expression results in increased susceptibility of TRICs to re-enter circulation, highlighting the crucial role of this regulatory axis in maintaining tissue residency.

### 3.2 Molecules mediating chemotaxis, migration, and adhesion

Tissue-specific adhesion molecules play distinct roles in different anatomical locations. CD11a serves as a crucial adhesion molecule in organs where CD103 expression is typically absent, such as the liver, spleen, and lymph nodes (19). Enhanced CD11a expression in hepatic tissue-resident CD8^+^T cells enables their maintenance within hepatic sinusoids (20). CD44, through its interaction with hyaluronic acid, facilitates both cell-matrix adhesion and indirect cell-cell interactions, while CD62L expression levels correlate with tissue localization capabilities, with CD62L^low^ cells preferentially forming tissue-resident populations (21, 22).

Chemokine receptors play a central role in guiding TRICs to specific tissues and maintaining their localization. CXCR6, a receptor for CXCL16, facilitates the migration and retention of TRIC precursors in peripheral tissues such as the liver, lungs, skin, and intestine (23, 24). In contrast, CCR7, which interacts with CCL19 and CCL21, promotes lymph node homing. Trm cells typically lack CCR7 expression, which prevents their recirculation to lymph nodes and supports their tissue residency (25, 26). The high CXCR6 and low CCR7 expression pattern is a hallmark of Trm cells, ensuring their retention and survival in peripheral tissues.

Tissue-specific chemokine receptors orchestrate precise TRIC localization through distinct molecular pathways. CCR5 is critical for maintaining mucosal barrier immunity, with up to 65% of CD4^+^Trm cells in mucosal tissues expressing high levels of CCR5, essential for their optimal positioning and function within the mucosal barrier (27). The CCR6-CCL20 axis mediates immune cell recruitment to inflammatory sites, with its disruption showing therapeutic potential in conditions like rheumatoid arthritis and inflammatory bowel disease (28). The CCR9-CCL25 pathway specifically promotes Trm cell differentiation and retention in gastrointestinal tissues, where these cells maintain local surveillance while remaining unresponsive to distant antigens (29). Similarly, CCR10-CCL27 signaling supports skin-specific immune responses, with CCR10^+^CD4^+^T cells being instrumental in mediating localized skin memory responses, particularly in conditions such as allergic contact dermatitis (30). This sophisticated chemokine receptor network ensures optimal tissue-specific immune surveillance and rapid response to local challenges.

### 3.3 Transcriptional regulators

Transcriptional regulation of tissue residency involves coordinated actions of key factors. Hobit and Blimp-1 collaboratively establish the tissue-resident program by suppressing circulation-associated genes (CCR7, S1PR1) while upregulating residence-related genes (CD69, CXCR6) (31, 32). Their tissue-specific roles vary, as evidenced in lung influenza-specific CD8^+^Trm cells, where Blimp-1, but not Hobit, is essential (33, 34). This suggests that Hobit and Blimp-1 employ distinct regulatory mechanisms across different tissues. Runx3 is another critical transcription factor that promotes the tissue-resident phenotype by upregulating CD103 expression (35). Overexpression of Runx3 enhances the persistence and effector functions of CD4^+^CAR-T cells, improving their tumor-residency capabilities (36). Additionally, Runx3 has been identified as a key regulator in tissue-resident T cells within healthy human breast tissue, highlighting its role in maintaining local barrier immunity (37).

### 3.4 Immune checkpoint molecules and other regulators

Inhibitory receptors (PD-1, TIM-3, LAG-3, TIGIT) and KLRG1 serve dual functions in TRICs (38, 39). While traditionally associated with T cell exhaustion, these molecules play crucial roles in maintaining tissue residency and preventing hyperactivation-induced tissue damage. For instance, PD-1 signaling promotes long-term memory T cell persistence by modulating metabolic programs (40, 41), while KLRG1 regulation through Bach2 facilitates the transition from effector to tissue-resident phenotypes (42, 43). Understanding this balance between inhibition and maintenance is crucial for therapeutic applications, particularly in cancer immunotherapy.

The development and maintenance of TRICs also depend on cytokine signaling networks. IL-15/IL-15Rα signaling is essential for TRIC formation and survival, promoting their persistence at infection sites (44). TGF-β signaling enhances the expression of tissue-residency markers such as CD103 and CD69, while suppressing circulation-associated genes. Mice lacking TGF-βR exhibit significant reductions in Trm cell numbers, highlighting its essential role in TRIC development (45). Additionally, RGS1, a negative regulator of chemokine receptor signaling, helps maintain tissue residency by limiting responsiveness to peripheral chemokine gradients, thereby reducing tissue egress (46).

## 4 Mechanisms of TRICs development and retention in tumor

**Figure 1:**
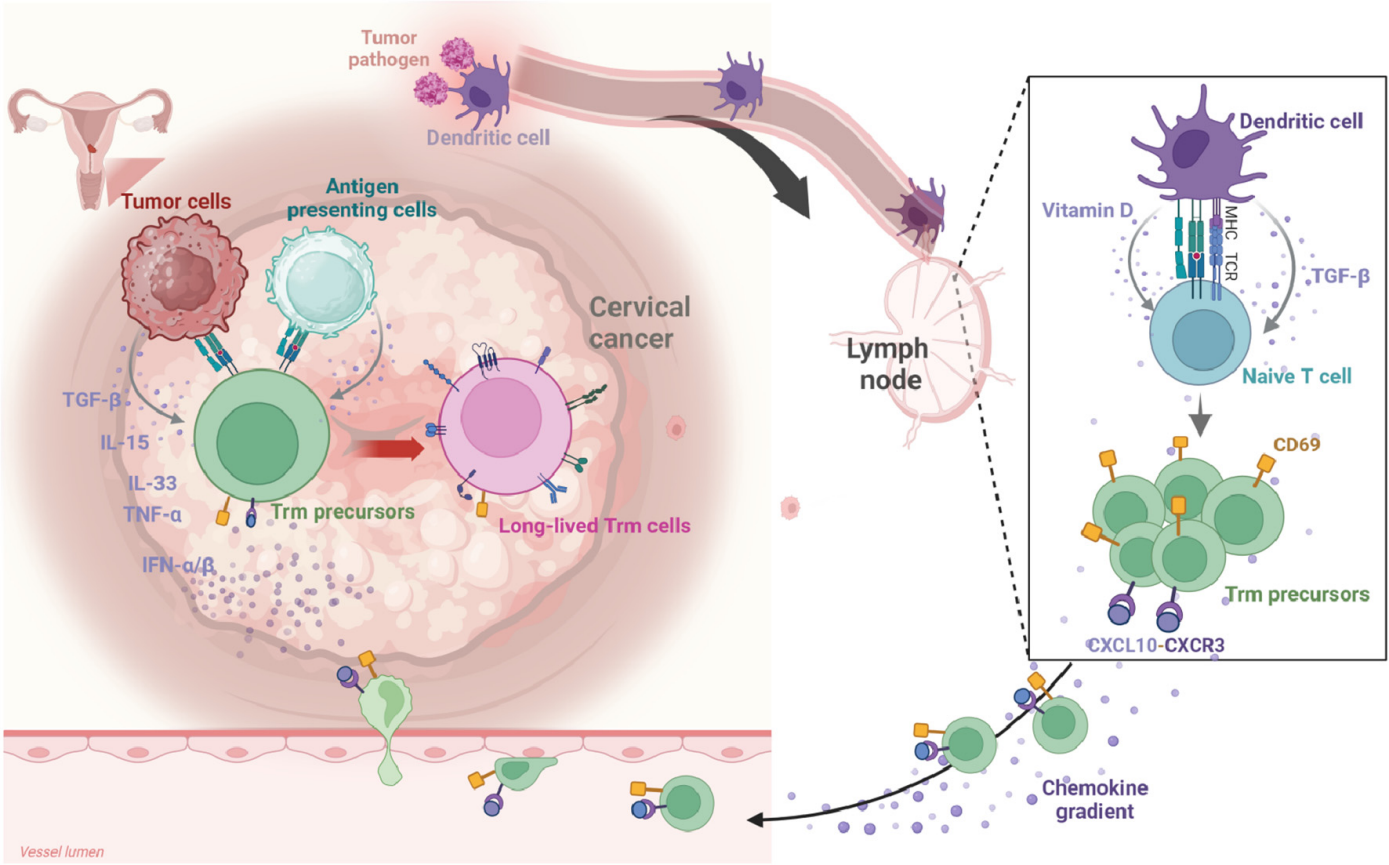
The development of Trm cells in CC. Migratory dendritic cells (DCs) capture tumor antigens within the tumor microenvironment (TME) and migrate to draining lymph nodes. There, they present these antigens to naïve T cells via MHC class molecules, initiating T cell activation and differentiation. Concurrently, DCs secrete cytokines, notably TGF-β, and metabolites like vitamins A and D, which influence T cell fate during the priming phase. Following activation, Trm precursors enter the arterial circulation, guided by chemokines (e.g., CCL5, CXCL9, CXCL10) that direct them toward tumor tissues. The upregulation of CD69 on Trm precursors inhibits S1PR1 signaling, preventing their return to circulation and anchoring them within the TME. Within this environment, Trm precursors undergo final differentiation, driven by a network of inflammatory cytokines and tumor antigen re-stimulation. Created in BioRender. Li, Q. (2025) https://BioRender.com/kfcf5cr.

Understanding the development and retention mechanisms of TRICs is crucial for advancing our comprehension of local immune surveillance and tumor immune responses. The molecular mechanisms governing TRIC biology remain largely unexplored. Currently, research on TRICs is predominantly concentrated on Trm cells, which serve as a model system for elucidating the fundamental principles of cellular tissue residency. This section will primarily focus on the detailed molecular mechanisms of Trm cell differentiation and retention (**Figure 1**), utilizing these insights as a framework to provide broader perspectives on TRIC development in the TME.

### 4.1 Priming and migration of Trm precursors

In tumor immunity, migratory dendritic cells (DCs) and specific subsets such as DC3 capture antigens within tumor tissues before migrating to draining lymph nodes, where they perform crucial antigen presentation functions (47–52). These DCs deliver tumor antigens to naïve T cells via MHC class I or II molecules, triggering T cell activation, proliferation, and functional differentiation. Concurrently, DCs secrete cytokines (particularly TGF-β) and metabolites such as vitamins A and D, which collectively regulate T cell fate determination during this priming phase. During activation, these signals not only stimulate naïve T cell proliferation but also induce expression of specific tissue-homing receptors. This molecular signature equips T cells with the capacity to migrate toward specific peripheral tissues, establishing favorable conditions for their subsequent differentiation into genuine Trm cells within the TME. Consequently, these “pre-programmed” T cells are considered Trm precursors, capable of rapidly converting into functional Trm cells upon receiving local environmental cues. 

Following lymph node activation, Trm precursors enter arterial circulation and achieve targeted homing in response to chemotactic signals (such as CCL5, CXCL9 and CXCL10) emanating from tumor or inflammatory sites (53, 54). Chemokines guide activated cells toward inflamed or tumor tissues by binding to corresponding receptors (such as CXCR3 and CCR5) on the cell surface. Ultimately, these Trm precursors traverse the endothelial barrier to enter tumor tissue. Prior to tissue entry, upregulation of surface CD69 plays a decisive role by inhibiting S1PR1 signaling, thereby preventing cells from returning to peripheral circulation and anchoring them within the local microenvironment.

### 4.2 Cytokine networks in Trm cell differentiation

In the tumor microenvironment, Trm precursor cells undergo a critical final differentiation phase orchestrated by a complex network of inflammatory cytokines and tumor antigen re-stimulation. This process is regulated by a diverse cellular ensemble including tumor cells, endothelial or epithelial cells, macrophages, and DCs that collectively establish a cytokine-rich milieu conducive to Trm development. TGF-β serves as the principal driver of Trm cell differentiation through multiple convergent pathways. Upon receptor binding, TGF-β activates the canonical Smad signaling cascade, whereby phosphorylated Smad2/3 forms complexes with Smad4 that translocate to the nucleus and directly promote CD103 gene transcription (55). Concurrently, TGF-β signaling induces critical transcription factors including Runx3 and Bhlhe40, which further reinforce Trm specific gene expression patterns (56, 57). In synergy with IL-33 and TNF-α, TGF-β also downregulates KLF2 through PI3K/AKT-dependent mechanisms, consequently reducing S1PR1 expression and preventing T cell egress from tissue sites. Additionally, the integration of TGF-β and Notch signaling further shapes the Trm phenotype. TGF-β upregulates Notch ligands on epithelial cells, enhancing Notch signaling in adjacent T cells. Activated Notch intracellular domain (NICD) translocates to the nucleus to regulate Hes1 and Hey1 expression, reinforcing Hobit and Blimp-1 expression while suppressing tissue egress-associated genes (S1PR1, CCR7, KLF2) (32).

IL-15 signaling complements TGF-β functions through interaction with its receptor complex (IL-15Rα/IL-2Rβ/γc), activating the JAK-STAT pathway to promote Trm differentiation and survival (58, 59). Phosphorylated STAT5 induces Hobit and Blimp-1 expression, while simultaneously engaging the PI3K/AKT axis to upregulate anti-apoptotic proteins Bcl-2 and Bcl-xL, substantially enhancing Trm resilience within the hypoxic, nutrient-deprived tumor microenvironment. A critical balance exists between IL-15 and TGF-β in regulating T-bet expression: IL-15 upregulates T-bet, thereby enhancing responsiveness to IL-15 itself through increased IL-15Rβ expression, while TGF-β moderately suppresses T-bet and Eomes to prevent excessive effector differentiation, simultaneously maintaining essential Trm characteristics. Consequently, mature Trm cells maintain T-bet at a “low but sufficient” level—adequate to ensure IL-15 signal sensitivity without compromising CD103 upregulation or allowing effector T cells to outcompete Trm populations.

Type I interferons (IFN-α and IFN-β) play a critical role in shaping Trm differentiation and function through multiple coordinated signaling pathways. These interferons not only upregulate chemokine receptors CXCR3 and CXCR6 via JAK-STAT signaling, thereby enhancing Trm positioning within the tumor microenvironment, but also induce IL-15 and IL-27 expression, creating a positive feedback amplification loop. Type I interferons additionally suppress KLF2 indirectly through PI3K/AKT pathways, reducing S1PR1 levels, while activating NF-κB signaling to upregulate adhesion molecules like CD49a, strengthening interactions between Trm cells and tumor matrix components. Additional cytokines further refine Trm differentiation and functional regulation through diverse pathways. IL-33 independently downregulates transcription of KLF2 and its target gene S1pr1 through PI3K/AKT-dependent mechanisms (17). This pathway converges with and amplifies similar signaling cascades initiated by type I interferons and TGF-β, establishing redundant mechanisms that ensure robust tissue retention. TNF-α predominantly enhances CD69 promoter activity through activation of NF-κB/Rel family members (particularly RelA), significantly increasing CD69 surface expression (60). This likely acts synergistically with adhesion molecules (such as CD49a) upregulated by type I interferons, collectively strengthening Trm cell anchoring within tumor tissues. Concurrently, IL-7 activates the Jak/STAT5 pathway to upregulate Mcl-1, Bcl-2, and Bcl-xL expression (61), forming a survival axis parallel to the IL-15-mediated PI3K/Akt-Bcl2 pathway, jointly maintaining long-term Trm cell persistence.

This multi-pathway cytokine signaling network ultimately converges on the coordinated activation of key transcription factors (including Blimp-1, Hobit, and RUNX3), driving significant upregulation of Trm signature molecules (CD103, CD49a, CXCR6, CD69) while simultaneously repressing circulation-associated gene expression (S1PR1, CCR7, CD62L) and certain effector-related transcription factors (high-level T-bet, Eomes). Through this multi-layered, cooperative signaling network regulation, precursor T cells within the TME complete their transformation into mature Trm cells, acquiring the capacity for long-term residence within tumor tissue.

The long-term maintenance of Trm cells in the TME is supported by a combination of cytokine signaling, metabolic adaptation, and epigenetic regulation. TGF-β and IL-15 remain critical for sustaining Trm survival and function, while hypoxia in the TME synergizes with TGF-β to stabilize the Trm phenotype. Metabolically, Trm cells rely on fatty acid oxidation (FAO) rather than glucose metabolism, enabling them to thrive in the nutrient-deprived, hypoglycemic conditions of the TME (62, 63). Transcription factors such as Bhlhe4 and Runx3 maintain the epigenetic landscape of Trm cells, ensuring the suppression of egress-related genes and the persistence of tissue-retention molecules like CD103 and CD69 (56, 64). Additionally, periodic antigen stimulation within the TME can enhance Trm proliferation and functionality, although excessive stimulation may lead to exhaustion (47).

## 5 Revisiting inhibitory receptors in TRIC cells: beyond exhaustion paradigms

Tissue-resident immune cells, particularly Trm cells, present a puzzling phenomenon in the tumor microenvironment: despite high expression of inhibitory receptors such as PD-1, TIM-3, LAG-3, and TIGIT, they maintain significant antitumor activity rather than exhibiting the expected functional exhaustion. This observation starkly contrasts with the conventional paradigm that views inhibitory receptors as hallmarks of T cell dysfunction and exhaustion. Understanding this paradox requires in-depth analysis across three dimensions: metabolic reprogramming, signaling pathway equilibrium, and transcriptional regulatory networks.

### 5.1 Metabolic reprogramming: PD-1-mediated survival advantage

The nutrient-restricted, lactate-rich TME presents significant survival challenges for conventional effector T cells. Under these conditions, the PD-1 signaling pathway unexpectedly confers protective benefits. Upon PD-1/PD-L1 binding, recruitment of SHP-2 and SHP-1 phosphatases inhibits the PI3K/AKT/mTOR pathway downstream of TCR signaling, shifting T cell metabolism from energy-intensive glycolysis toward more economical fatty acid oxidation (FAO) (40, 65). Specifically, PD-1 signaling upregulates carnitine palmitoyltransferase 1A (CPT1A) expression, enhancing FAO while simultaneously attenuating glycolysis and glutaminolysis (66). This metabolic shift particularly benefits Trm cells for several reasons: it delays rapid apoptosis that would otherwise occur in glucose-deprived environments due to excessive glycolytic dependence; it prevents terminal differentiation triggered by high-intensity metabolic activity in effector T cells; and most importantly, it establishes a metabolic profile conducive to long-term survival, thereby maintaining persistent immunosurveillance within the TME. Thus, high PD-1 expression on Trm cells likely represents an adaptive mechanism rather than a marker of functional impairment.

### 5.2 Signaling pathway balance: antagonistic regulation by IL-15 and TGF-β

Maintenance of Trm functionality depends on a sophisticated cytokine network, particularly the balance between IL-15 and TGF-β. IL-15 activates STAT5, enhancing IFN-γ gene transcription and moderately upregulating T-bet expression. STAT5 directly binds to both the proximal promoter and the CNS1 enhancer of the IFNG gene, while T-bet directly promotes IFNG transcription. Conversely, TGF-β, through the Smad signaling pathway, suppresses high-level expression of T-bet and Eomes, preventing excessive acquisition of effector phenotypes while inducing expression of tissue retention molecules such as CD103. This antagonistic balance is crucial for Trm function: T-bet is maintained at low but sufficient levels (53, 67, 68), which both ensures Trm sensitivity to IL-15 survival signals through upregulation of IL-15Rβ (CD122) and avoids the inhibitory effect of high T-bet levels on Trm formation. Notably, reduced T-bet levels weaken suppression of inhibitory receptors such as PD-1, resulting in increased expression of these molecules without completely blocking effector functions—a key mechanism potentially explaining how Trm cells maintain functional activity despite high inhibitory receptor expression.

### 5.3 Microenvironmental adaptation: hypoxia and TGF-β synergy

Hypoxic conditions within the TME activate HIF-1α, which enhances TGF-β signaling, establishing another critical regulatory mechanism. HIF-1α not only promotes glucose uptake and metabolism but also potentiates TGF-β/Smad signaling pathway activity. This synergistic interaction reinforces tissue-resident characteristics of Trm cells while maintaining their survival capacity in hypoxic environments (69). Hypoxia-driven metabolic adaptation intersects with PD-1 signaling: PD-1-mediated metabolic shifts favor long-term cellular survival under hypoxic conditions, while interactions between HIF-1α and TGF-β signaling further stabilize the Trm phenotype (70). This multilayered regulation enables Trm cells to maintain sufficient functional activity in response to tumor antigen stimulation despite high inhibitory receptor expression.

Furthermore, after anti-PD-1 treatment, the transcriptional characteristics of Trm cells undergo significant changes, exhibiting functional states similar to effector memory T cells (71). This further suggests that Trm cells expressing inhibitory receptors are not in an irreversible exhausted state, but rather in a highly plastic functional state. However, sustained PD-1 blockade may yield dichotomous consequences: initial function restoration and enhancement, followed by potential terminal differentiation due to intensified glycolysis, ultimately leading to clonal deletion. This phenomenon offers a mechanistic explanation for the observed patterns of therapeutic resistance in clinical settings, where patients initially responding to PD-1 blockade subsequently develop progressive disease (72, 73). The temporal dynamics of Trm functional modulation are characterized by initial reinvigoration followed by potential terminal differentiation and deletion, which closely align with clinical response kinetics. Moreover, this biphasic response pattern suggests that intermittent rather than continuous checkpoint blockade might better preserve the Trm population while maintaining antitumor efficacy.

Despite relatively limited research on tissue-resident immune cells beyond Trm cells, emerging studies have identified high expression of inhibitory receptors in other TRIC subtype like tissue-resident NK cells (74). This observation suggests that similar functional equilibrium and adaptive mechanisms may operate in these cell populations, extending the paradigm of inhibitory receptor-mediated homeostasis across diverse tissue-resident lymphocyte lineages. The phenomenon of TRIC cells maintaining functional activity despite high inhibitory receptor expression reflects an exquisite biological equilibrium. Within this balance, inhibitory receptors serve not merely as functional “brakes” but as “stabilizers” for adaptation to harsh TME conditions. Through these receptors, Trm cells modulate their metabolic state and signal intensity, establishing a balance between terminal differentiation and functional maintenance, thereby achieving long-term residence and preserved functionality within nutrient-depleted, hypoxic, and highly suppressive tumor microenvironments.

## 6 Tissue-resident memory CD8^+^T cells in CC: the predominant tumor-reactive population

CD8^+^Trm cells represent a critical frontier in understanding anti-tumor immunity within CC. Strategically positioned within tumor tissues, these specialized immune sentinels offer a unique mechanism of immune surveillance that transcends traditional circulating T cell responses. Their distinctive molecular profile provides key insights into the complex immunological dynamics of CC progression.

### 6.1 Molecular markers and phenotypic characteristics

CD8^+^ tissue-resident memory T cells (CD8^+^Trms) play a crucial role in anti-tumor immunity within CC microenvironments. Using single-cell RNA sequencing techniques, researchers have conducted comprehensive analyses of Trm cells in CC tissues. Beyond the well-established core markers CD103 and CD69, CD8^+^Trm cells of CC express significantly higher levels of tissue-residence-associated genes (such as TGFB1 and ITGB7), cytotoxicity genes (including GZMB and PRF1), chemokine genes (notably CXCL13 and CCL5) and inhibitory molecules (LAG3, TIGIT, PDCD1, HAVCR2, and CTLA4), compared to non-Trm cells (75). This distinct transcriptional profile reflects their central role in tumor immune surveillance.

### 6.2 Clinical prognostic relevance

A large-scale epidemiological study has demonstrated significantly higher diagnosis rates and mortality from advanced CC among women aged over 65 years compared to younger populations (76, 77). This age-related disparity in clinical outcomes correlates with the progressive decline in cervical Trm cells observed in aging populations (78). The depletion of cervical Trm cells in postmenopausal women may represent a crucial mechanism underlying the poor prognosis in elderly CC patients. Notably, CD8^+^Trm cell density has emerged as a key prognostic indicator across various solid tumors, correlating with tumor size, grade, and overall survival (79–83).

Studies have shown that the expression of the ITGAE gene (encoding CD103) significantly correlates with T cell markers (CD3, CD2), immune checkpoint molecules (PD1, TIGIT), and antigen presentation molecules (HLA-DR, HLA-DQ) (84). High CD103 expression is associated with significantly improved cancer-specific survival, a correlation that remains statistically significant after adjusting for disease stage. Recent investigations have demonstrated that infiltration levels of CD103^+^CD8^+^tumor-infiltrating lymphocytes strongly correlate with improved survival outcomes in CC patients (78). This finding has been robustly validated in an independent cohort of 460 CC patients, establishing the expression of these cells as an independent predictive factor for patient prognosis. Furthermore, CD8^+^Trm cell infiltration is substantially higher in CC tissues compared to normal cervical tissues (75, 85), highlighting their critical role in anti-tumor immune responses.

### 6.3 Spatial distribution characteristics

Recent spatial transcriptomic analyses of CC have revealed three distinct patterns of CD8+ T cell distribution: the infiltrated pattern, where CD8^+^T cells effectively penetrate and distribute throughout the tumor epithelium; the excluded pattern, characterized by CD8^+^T cells predominantly accumulating in the peritumoral stroma without entering the tumor parenchyma; and the desert pattern, marked by an almost complete absence of CD8^+^T cell infiltration. Importantly, the infiltrated pattern strongly correlates with the MP7 tumor state (epithelial-immune phenotype), which is characterized as a prototypical “immune-hot” tumor where tumor cells highly express antigen-presenting molecules (HLA-DR, HLA-DQ), immune response genes (B2M, CXCL10), and immunomodulatory factors (IDO1, CD274), indicating active antigen presentation and sustained immune activation (86).

CD8^+^T cells within MP7 regions concurrently express elevated levels of cytotoxic effector molecules (GZMB, PRF1, GNLY) and inhibitory receptors (HAVCR2, TIGIT, LAG3, CTLA4, PDCD1). This molecular signature shows remarkable concordance with CD8^+^Trm cell markers identified in single-cell sequencing study (75), suggesting that CD8^+^T cells infiltrating the tumor epithelium are predominantly CD8^+^Trm subpopulations, further validating the critical role of CD8^+^Trm cells in immune-hot tumor microenvironments. Spatial interaction analyses further demonstrate that MP7 tumor cells likely actively recruit CD8^+^T cells to epithelial regions through chemokine-receptor axes, including CXCL9–CXCR3 and CXCL11–CXCR3. Additionally, PROGENy pathway enrichment analysis reveals significant activation of JAK-STAT, MAPK, and NF-κB signaling in the MP7 state, pathways involved in regulating pro-inflammatory cytokine production and immune cell recruitment, establishing the molecular basis for the preferential enrichment of CD8^+^Trm cells within tumor epithelial regions.

### 6.4 Molecular regulatory mechanisms of epithelial affinity

The epithelial tropism of CD8^+^Trm cells is regulated by intricate molecular mechanisms. CD103, a critical adhesion molecule, requires dual signaling for its expression: T cell receptor (TCR) activation and TGF-β signaling. Research by Komdeur et al. revealed that CC exhibits a distinctive TGF-β signaling profile compared to other malignancies (78). In CC, TGF-β signaling is remarkably abundant throughout the tumor microenvironment, evidenced by prominent nuclear pSMAD2/3 expression in tumor nests, surrounding stromal cells, and both CD103^+^ and CD103^-^tumor-infiltrating lymphocytes. The characteristic distribution pattern of TGF-β signaling closely relates to HPV biology, as HPV-16 E6 and E7 oncoproteins directly modulate TGF-β1 expression in cervical tumor cells through specific DNA sequence motifs in the TGF-β1 core promoter. Within this TGF-β-enriched microenvironment, direct contact between T cells and tumor cells becomes the decisive factor for CD103 induction, positioning CD103 as an ideal biomarker for identifying tumor-reactive T cells in CC. Beyond TGF-β signaling, as previously described (86), the MP7 tumor state in CC features activated signaling pathways such as JAK-STAT and MAPK that form complex regulatory networks with immune cell recruitment, collectively shaping the tissue localization properties of CD8^+^Trm cells in CC.

### 6.5 Cell dynamics in disease progression

Single-cell sequencing analysis has revealed significant gene expression differences in CD8^+^T cells across various stages of CC progression (87). In early-stage CC, CD8^+^T cells exhibit robust anti-tumor immune characteristics, marked by highly activated interferon signaling pathways (JAK2, STAT1, IFIH1, ISG15, GBP1, GBP4, GBP5, OAS1, OAS2, OAS3) and enhanced cytotoxic functionality (GZMB, PRF1). Notably, these cells also upregulate RUNX3, CXCR6, CXCL13, and CCL5, a molecular signature strikingly similar to the established profile of Trm identified in previous studies. This suggests that CD8^+^Trm cells likely constitute the predominant anti-tumor CD8^+^T cell population in early CC, strategically positioned for immediate immune surveillance and rapid response to tumor antigens.

As the disease progresses to advanced stages, the molecular profile of CD8^+^T cells undergoes substantial transformation. The previously upregulated immune activation genes show markedly reduced expression levels, replaced by genes associated with tissue remodeling (COL1A1, COL1A2, COL3A2, CYBA, HLA-DPA1) and immunosuppression (S100A8, S100A9). This shift reflects a transition from a highly effective anti-tumor state toward a tissue-adaptive, functionally limited state, likely connected to tumor microenvironment remodeling and immune evasion mechanisms.

Recent spatial transcriptomic analysis of CC has identified distinct tumor molecular phenotypes that correlate with specific immune infiltration patterns. Two predominant states have emerged: MP7, an immune-active tumor state corresponding to the CD8^+^T cell infiltration pattern within tumor epithelium; and MP6, an immune-suppressive tumor state characterized by high expression of keratinization-associated genes (FABP5, SERPINB3, S100A8/9) and overactivation of the TGF-β signaling pathway. MP6-dominant tumors display “excluded/desert” immune phenotypes, where CD8^+^T cells are largely prevented from entering tumor epithelial regions. These tumor states correspond to specific spatial distribution patterns of immune cells (infiltrated or excluded/desert pattern), which directly impact anti-tumor immunity and treatment response.

Comparing results from these CC single-cell and spatial transcriptomic studies reveals a striking parallel in molecular characteristics: CD8^+^T cells in early-stage CC show high similarity in gene expression profiles to CD8^+^T cells in the MP7 tumor state, while CD8^+^T cells in late-stage CC exhibit significant commonalities with those in the MP6 tumor state. This correspondence illuminates the tight coupling mechanism between the tumor immune microenvironment and T cell functionality.

This dynamic evolution of CD8^+^T cell phenotypes, coupled with the corresponding tumor state transitions, provides critical insights into the mechanisms of immune surveillance and escape during CC progression. The early dominance of CD8^+^Trm cells in MP7-associated “hot” tumors gives way to immune exclusion in MP6-associated advanced tumors, highlighting potential therapeutic targets for different disease stages.

### 6.6 CD8^+^Trm dynamics in CC treatment

Notably, patients undergoing chemoradiotherapy typically exhibit lower CD103^+^Trm infiltration compared to those treated with surgery alone (78). This difference stems from the fact that robust CD103^+^Trm infiltration signifies an immune-active tumor state, where potent anti-tumor immunity effectively restricts tumor growth, often corresponding to early-stage disease. In such cases, surgery is the preferred treatment option. Conversely, tumors with minimal CD103^+^Trm infiltration, indicative of an immune-suppressed microenvironment, are more likely to be advanced and thus require chemoradiotherapy for disease control.

Single-cell RNA sequencing provided unprecedented insights into the time-dependent immunological remodeling mechanisms following chemoradiotherapy in CC (88).At three weeks post-treatment, while the absolute number of CD103^+^ Trm cells temporarily decreased, the tumor microenvironment underwent a series of changes favorable to subsequent immune activation. Most notably, tumor epithelial cells upregulated MHC-II expression, significantly enhancing their antigen presentation capabilities. Concurrently, chemoradiotherapy promoted CD16^+^ NK cell infiltration and increased their cytotoxicity-related gene expression (GZMB, GZMH, NKG7). Moreover, the proportion of exhausted CD8^+^ T cells significantly declined, while CD4^+^ naive T cell proportions increased.

These findings reveal a specific “immune intervention window” at three weeks post-treatment, where innate immune systems are activated, but adaptive immune responses have not yet fully developed. During this period, tumor cells release increased antigens with enhanced presentation capabilities and reduced microenvironmental immunosuppression, creating ideal conditions for targeting HPV-specific T cell activation. Preclinical research provided compelling evidence for leveraging this post-chemoradiotherapy immune window (78). Combining therapeutic vaccination using forest virus expressing E6/E7 with radiotherapy synergistically elevated CD8^+^CD103^+^T cell proportions from approximately 10% in untreated tumors to 60% in the combined treatment group. This strategy effectively exploited the “danger signals” generated by chemoradiotherapy alongside increased tumor antigen exposure, thereby enhancing vaccine-mediated anti-HPV adaptive immune responses.

## 7 Tissue-resident memory CD4^+^T cells in CC: the key accessory cells for CD8^+^Trm cells?

While the role of CD8^+^Trm cells in antitumor immunity is well established, the functional significance of CD4^+^Trm cells in CC remains less understood and somewhat controversial. Comprehensive single-cell RNA sequencing analyses across multiple cancer types have revealed that tumor-infiltrating Trm cells are predominantly CD8^+^T cells, characterized by the expression of tissue residency and cytotoxicity-associated genes. In contrast, CD4^+^T cells within the tumor microenvironment exhibit limited expression of functional activation markers, such as IFIT3, suggesting potential functional impairment (89).

However, emerging evidence challenges this perspective. Studies utilizing TCRα-deficient mouse models have demonstrated that CD4^+^Trm cells, marked by CXCR6 expression, play a critical role in initiating antitumor immune responses. These cells secrete IFN-γ, which triggers NK cell-mediated tumor elimination through a coordinated immune cascade. These findings highlight the complexity of CD4^+^Trm cell functions within the tumor microenvironment, where they may act as essential collaborators in enhancing overall tumor immunity by facilitating cytokine secretion and promoting the activation of other immune cells (90).

Recent investigations have demonstrated that CD4^+^CD103^+^T cells are the predominant subset producing pro-inflammatory cytokines IFN-γ and TNF-α, with their numbers significantly increased in tumor tissues compared to normal tissues. These cells exhibit a distinctive molecular signature, including high expression of inhibitory receptors like PD-1 and TIM-3, and the tissue-resident marker CD103 (91). Single-cell sequencing analysis of CC has shown significant clonal expansion of CD4^+^CXCL13^+^T cells within tumor tissues, indicating antigen-driven local proliferation (92). Although the tissue residency status and functional significance of these expanded clones remain to be fully characterized, insights from melanoma research suggest that CD4^+^CXCL13^+^T cells display tumor antigen specificity and express a distinctive molecular signature, including the inhibitory receptor TIM-3, tissue-resident marker CD103, and effector cytokine IFN-γ. Importantly, the frequency of these cells shows a positive correlation with CD8^+^PD1^+^CXCL13^+^T cell infiltration (93), suggesting a coordinated immune response.

These findings collectively suggest that CD4^+^Trm cells function as critical orchestrators of antitumor immunity through multiple mechanisms. Their local cytokine production appears to support CD8^+^T cell function, while CXCL13 secretion likely facilitates immune cell recruitment and organization within the tumor microenvironment. This coordinated interaction between CD4^+^ and CD8^+^Trm cells may represent a crucial mechanism for effective antitumor immunity, with significant implications for developing more effective immunotherapeutic strategies.

## 8 Tissue-resident B cells in CC: the potential tumor antigen-specific B cell?

While B cell presence in mucosal tissues has been documented for over two decades (94–97), the formal identification of tissue-resident B cells was not established until 2019, through definitive experiments employing parabiosis and FTY720 blockade (9). Current research has predominantly focused on lung tissue-resident memory B (Brm) cells, which are characterized by high expression of CD69 and specific chemokine receptors, notably CCR6 and CXCR3 (98–100). Mechanistically, CCR6 has been demonstrated to facilitate Brm cell retention within human bronchial-associated lymphoid tissues (100). These cells primarily function to mount rapid, localized humoral responses against respiratory pathogens, including influenza virus.

The discovery of tissue-resident B cells across various organs, including skin, intestines, lungs, and uterus, has sparked interest in their potential roles in tumor immunity. This interest has been further intensified by the established correlation between TLS and favorable clinical outcomes across multiple cancer types. Tumor-infiltrating B cells (TIL-Bs) typically exist within complex immune cell networks, interacting with T cells, NK cells, and other immune components. Notably, their presence is particularly pronounced in highly immunogenic “hot” tumors, where CD4^+^ and CD8^+^ Trm cells express elevated levels of CXCL13, the key B cell recruitment factor. 

Recent comprehensive analyses across various cancer types, including colon, lung, and kidney cancers, have revealed that memory B cells constitute the predominant TIL-B subset (101). These cells are characterized by high expression of ITGA4, which facilitates their entry into peripheral tissues (102, 103). The persistent presence of tumor antigens appears to drive continuous recruitment and local expansion of memory B cells, maintaining their sustained presence within the tumor microenvironment. One antigen receptor lineage analyze has demonstrated that TIL-Bs exhibit distinct memory B cell characteristics with evidence of localized expansion and differentiation, particularly in breast cancer tissues (104).The presence of memory B cell subsets within tumor tissues positively correlates with improved overall and disease-free survival rates in multiple cancer types, including CC, sarcoma, cutaneous melanoma, and endometrial cancer (93).In lung cancer, CD69^+^TIL-Bs demonstrate enhanced antigen presentation capabilities to CD4^+^TILs, modulating their phenotype and function (93).

A recent single-cell transcriptomic analysis of CC patients has revealed distinct B cell populations with tissue-resident characteristics (92). Notably, a CD69^+^B cell population expressing tissue-residency and memory-associated genes (CCR6, ITGA4, ITGB7, and CD80) was identified, showing enrichment in antigen processing and presentation pathways. Additionally, a distinct CD103^+^B cell population was observed within well-organized TLS, correlating with enhanced CD8^+^T cell infiltration and improved clinical outcomes. Trajectory analysis suggests a potential differentiation pathway from CD69^+^B cells to CD103^+^B cells, marked by dynamic changes in gene expression patterns, including CCR6 downregulation and Bcl6 upregulation, potentially reflecting their functional evolution from antigen processing to specific anti-tumor responses.

The observed developmental trajectory between CD69^+^ and CD103^+^ B cells represents a critical paradigm in understanding B cell-mediated anti-tumor immunity. This transition appears to be orchestrated by a complex interplay of transcriptional regulators and microenvironmental signals. The downregulation of CCR6, traditionally associated with tissue retention and trafficking, coupled with the upregulation of Bcl6, a master regulator of germinal center formation, suggests a programmed transition from tissue surveillance to active participation in local immune responses. This phenotypic evolution may be driven by persistent exposure to tumor antigens and local inflammatory signals within the tumor microenvironment.

## 9 Tissue-resident NK cells in CC: immune regulators rather than tumor killers?

NK cells represent a distinct arm of innate immunity, characterized by their ability to eliminate tumor cells and virus-infected cells without prior antigen sensitization, positioning them as crucial first-line defenders in anti-tumor immunity (105, 106). Their activation is primarily regulated through a sophisticated balance between inhibitory signals, predominantly mediated by MHC-I recognition, and activating signals. This unique regulatory mechanism enables NK cells to effectively target tumor cells that have downregulated MHC-I expression as an immune evasion strategy, thereby complementing T cell-mediated immunity (107). In CC specifically, NK cell infiltration correlates positively with neoadjuvant chemotherapy efficacy and improved clinical outcomes (108).

CD56^bright^NK cells emerge as the predominant tissue-resident NK subset across lymphoid and peripheral tissues, demonstrating significant involvement in local tumor immunity (109). In uterine tissue, these cells comprise 30-70% of the total NK cell population, characterized by a CD56^bright^CD49a^+^phenotype (109, 110). The tissue-resident signature of these cells is further defined by the expression of specific markers including CD69, CD103, ITGβ7, and CXCR6 (109–111). Functionally, uterine tissue-resident CD56bright NK cells exhibit a unique profile characterized by reduced cytotoxicity but enhanced cytokine production capacity.

Recent comprehensive single-cell transcriptomic analysis across 24 cancer types has revealed distinct functional profiles of CD56^bright^CD16^lo^ and CD56^dim^CD16^hi^NK cells (112). CD56^dim^CD16^hi^NK cells demonstrate elevated expression of cytotoxic effector molecules, including perforin and various granzymes (GZMB, GZMA and GZMH). In contrast, CD56^bright^CD16^lo^NK cells express diverse cytokine genes, particularly IL-8, indicating their prominent role in immune regulation through cytokine production (113, 114). A notable discovery is the exclusive expression of GZMK in CD56^bright^CD16^lo^NK cells (112), paralleling the identification of GZMK^+^CD8^+^T cells as a specialized tissue-resident subset associated with improved survival in various cancers (115, 116).

The pan-cancer analysis has identified RGS1 as a novel marker highly expressed in tumor-associated CD56^bright^NK cells but absent in circulating NK cells. The combination of RGS1 and CD69 provides superior discrimination between tissue-resident and circulating NK cells compared to conventional markers alone. Mechanistically, RGS1 expression may promote tissue residency through attenuation of G protein-mediated chemotactic signaling (112). While research on tissue-resident NK cells in CC remains limited, recent single-cell sequencing has identified a distinct CD103^+^GZMK^+^NK cell population enriched within tumor tissues. Analysis of TCGA data reveals that high expression signatures of this subset correlate with improved overall survival (92), suggesting a potentially crucial role for tissue-resident NK cells in CC immunity.

In the context of CC, the enrichment of CD103^+^GZMK^+^NK cells and their correlation with improved survival outcomes raises several important questions. First, what factors within the CC microenvironment promote the accumulation and maintenance of these cells? Second, how do these tissue-resident NK cells interact with other components of the local immune system, particularly tissue-resident T cells and antigen-presenting cells? Understanding these interactions could reveal new therapeutic opportunities and improve current immunotherapy strategies.

## 10 Therapeutic strategies targeting TRICs in CC

Emerging immunotherapeutic approaches offer new perspectives and possibilities for CC treatment, despite the limitations of traditional surgical, radiotherapeutic, and chemotherapeutic interventions in advanced and recurrent cases. By integrating recent immunotherapy advancements and a comprehensive understanding of tissue-resident immune cells, this manuscript explores innovative targeted therapeutic strategies that may revolutionize CC management.

Immune checkpoint inhibitors have demonstrated remarkable efficacy across multiple cancer types by disrupting immunosuppressive pathways and activating T cell-mediated tumor cell elimination (117). In CC treatment, checkpoint inhibitors such as pembrolizumab and nivolumab have been applied to advanced and recurrent patients, yielding preliminary clinical benefits (118, 119). However, monotherapy remains constrained by limited response rates, with many patients experiencing initial treatment effectiveness followed by progressive disease and immune resistance. Considering the critical role of CD8^+^Trm cells in CC and the profound impact of high PD-1 expression within the hostile late-stage tumor microenvironment, we propose a novel hypothesis. During initial treatment, Trm cells exhibit effector-like characteristics due to PD-1 signal blockade, yet the maintenance of their effector functions critically depends on sufficient energy supply. The metabolically restricted late-stage tumor environment ultimately compromises these activated Trm cells, leading to functional exhaustion and apoptosis.

Based on our theoretical understanding of Trm cell metabolism and immune checkpoint dynamics, we propose potential strategic approaches that warrant further investigation to enhance immune checkpoint inhibitor responsiveness. Preliminary hypotheses suggest that implementing intermittent anti-PD-1 treatment may alleviate local tumor metabolic burden, potentially enabling T cells to maintain long-term survival through fatty acid oxidation during metabolic stress periods. Additionally, we speculate that combining anti-PD-1 therapy with metabolic environment-modulating agents could potentially offer novel therapeutic strategies. Specifically, agents like metformin, which decrease tumor hypoxia (120, 121), and low-dose bevacizumab, which promotes vascular normalization, might provide innovative approaches to modulating the tumor microenvironment and enhancing immunotherapeutic efficacy (122). However, these approaches remain purely theoretical and require rigorous experimental validation and comprehensive clinical trials to establish their efficacy and safety in cervical cancer treatment.

Immunocellular therapies, including CAR-T cell and tumor-infiltrating lymphocyte (TIL) approaches, offer innovative strategies for directly activating and engineering patient-derived immune cells to enhance antitumor capabilities. TIL therapy, involving extraction, ex vivo expansion, and reinfusion of tumor-infiltrating immune cells, has demonstrated preliminary success in CC treatment. HPV-targeted adoptive T cell therapies have garnered particular attention. One study involving nine patients showed that two cases complete responses at 15 and 22 months post-treatment (123).

CAR-T cell therapy represents a sophisticated genetic engineering approach that introduces chimeric antigen receptors into patient T cells, enabling precise tumor cell recognition and elimination. Given the high expression and stability of HPV E6 and E7 proteins in CC, these antigens have emerged as critical CAR-T therapeutic targets. Preclinical mouse models demonstrated that HPV E7-targeted CAR-T cells successfully induced CC regression, establishing fundamental tumor degradation mechanisms (124). A phase I/II clinical trial targeting HPV-16-positive patients demonstrated the safety and preliminary efficacy of E7-targeted CAR-T cell therapy, with partial tumor remission observed in select patients (125).

Recognizing the unique advantages of CD8^+^Trm cells—including their tumor antigen-specific killing potential and capacity for sustained tissue residency—targeted immunotherapies focusing on tissue-resident immune cells present extraordinary potential for advanced and metastatic CC treatment. This innovative therapeutic strategy transcends traditional T cell engineering, emphasizing the distinctive biological characteristics of TRICs, such as their persistent presence and specific recognition capabilities within the tumor microenvironment. While targeting TRICs offers a promising new direction in CC immunotherapy, significant challenges remain. The complex acquisition and expansion of tissue-resident immune cells, high CAR-T cell production costs, incomplete understanding of molecular mechanisms, and the intricate process of translating laboratory discoveries into clinical applications necessitate continued rigorous research and interdisciplinary collaboration.

## 11 Discussion

**Figure 2:**
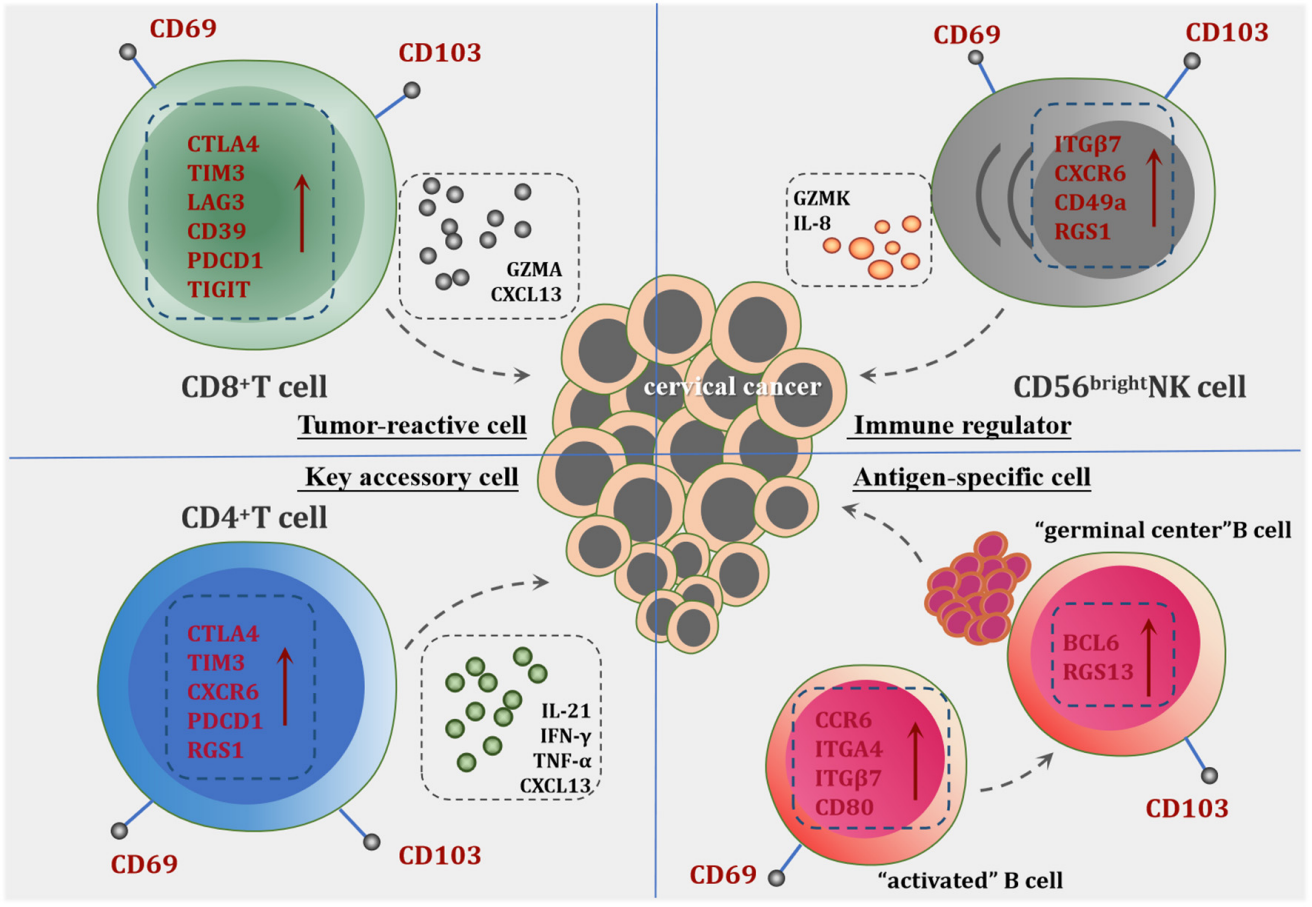
The potential role of tissue-resident immune cells in CC. CD8^+^Trm cells are characterized by high expression of inhibitory receptors and activation markers, including CD39, LAG3, PD-1 (PDCD1), TIM-3, and TIGIT, indicating their tumor antigen specificity and activation status. They demonstrate significant local expansion within tumor tissues, contributing directly to anti-tumor immunity through cytotoxic activity against tumor cells. CD4^+^Trm cells play a pivotal role in supporting CD8^+^Trm cells by the release of cytokines such as IFN-γ, TNF-α and chemokine CXCL13, which facilitate the recruitment and activation of other immune cells. Tissue-resident NK Cells exhibit robust cytokine production capabilities, participating in tumor immune regulation. The significant upregulation of GZMK distinguishes them from conventional NK cells, underscoring a unique functional profile that may contribute to enhanced anti-tumor responses. Tissue-resident B Cells are categorized into CD69^+^B cells and CD103^+^B cells. The presence of the CD69^+^B cell subset indicates the acquisition of local residency and memory capabilities within tumor tissues. The differentiation tendency of CD69^+^B cells toward CD103^+^B cells suggests heightened specificity for tumor antigens and a substantial role in TLS formation, thereby amplifying the humoral immune response against the tumor.

In this review, we have provided a comprehensive overview of tissue-resident immune cells (TRICs) in cervical cancer (CC), highlighting their potential characteristic markers and functional roles in tumor immunity (**Figure 2**). While significant progress has been made in understanding these tissue-resident populations, several critical challenges remain to be addressed. First, the prognostic value of tissue-resident immune cells and their correlation with immunotherapy outcomes warrant further investigation through large-scale clinical studies. Understanding how these immune cells influence patient responses to therapies will be essential for developing personalized treatment strategies. Second, the molecular mechanisms governing the differentiation and maintenance of tissue-resident immune cells within the CC microenvironment remain largely undefined. Of particular importance is the identification of key factors that regulate their tissue-residency programs. Future research should focus on developing strategies to enhance the tissue-resident properties of tumor-infiltrating immune cells and increase their density within CC tissues. These advances will be crucial for designing novel therapeutic approaches targeting tissue-resident immune cells.

Moreover, CD8^^+^^Trm cells represent a core population in the immunobiology of CC, linking HPV-specific adaptive immunity to clinical outcomes. Their unique tissue-residency characteristics, functional adaptability, and therapeutic operability position them as central players in natural tumor control and immunotherapy-mediated responses. A deeper understanding of the heterogeneity of Trm subsets, their tissue localization patterns, and the cellular interaction networks will provide new directions for developing targeted immunotherapeutic strategies.

To further expand the discussion on future research directions, we propose several key areas for investigation. There is a pressing need to identify biomarkers that can predict Trm-mediated immune responses, as these biomarkers could facilitate more precise patient stratification for tailored therapies, enhancing the effectiveness of immunotherapy in cervical cancer. Future studies should also explore the interactions between different immune cell subsets within the tumor microenvironment, as understanding how Trm cells communicate with other immune cells, such as dendritic cells, macrophages, and other T cell populations, will be crucial for elucidating the complex immune landscape in cervical cancer. Investigating combination therapies that integrate checkpoint inhibitors with strategies aimed at enhancing Trm cell function could yield promising results; for instance, exploring how metabolic interventions can restore the effector functions of Trm cells in advanced cervical cancer may provide new therapeutic avenues. Additionally, research should focus on optimizing strategies to exploit the immunological window following chemotherapy and radiotherapy, as understanding the timing and conditions under which Trm cells can be effectively mobilized or expanded will be critical for improving patient outcomes. Lastly, there is a need to develop vaccines or cell therapies that can specifically induce and expand HPV-specific Trm cells, as such approaches could enhance the immune response against cervical cancer and improve long-term patient survival.

By addressing these areas, future research can pave the way for comprehensive immunotherapy strategies that leverage the unique properties of tissue-resident immune cells in cervical cancer. Ultimately, a deeper understanding of these aspects may lead to more effective immunotherapy strategies and improved clinical outcomes for CC patients.
